# What should convince a clinician of disease modification in Alzheimer's disease clinical trials?

**DOI:** 10.1002/alz.71643

**Published:** 2026-07-01

**Authors:** Jemma Hazan, Kathy Y. Liu, Robert Howard

**Affiliations:** ^1^ Division of Psychiatry University College London London UK; ^2^ North London NHS Foundation Trust London UK

**Keywords:** Alzheimer's disease, amyloid‐lowering, clinical trial, dementia, disease modification

## Abstract

The recent publication of extension data from post‐regulatory trials of amyloid‐lowering antibody therapies, such as donanemab and lecanemab, requires clinicians to critically appraise trial designs and analyses in order to evaluate claims made by authors and sponsors of disease modification. This Perspective outlines the challenges with assessing claims of disease course modification, provides a framework for their evaluation, reviews the different trial designs and analytical approaches used to test or support them, and proposes a practical appraisal checklist for evaluating studies.

## Highlights

- Delayed‐start trials provide a robust design to assess Alzheimer's disease (AD) disease modification.
- Pre‐specified disease‐modification hypotheses should be included in trials.
- Clear reporting of estimands, outcomes data, and attrition is essential.

## 1 INTRODUCTION

Alzheimer's disease (AD) therapeutics have entered a new phase, with three amyloid‐targeting monoclonal antibodies now having received regulatory approval. In the United States, aducanumab received accelerated approval in 2021,^1^ followed by lecanemab in 2023,^2^ and donanemab was approved in 2024,^3^ via the standard regulatory pathway. In the United Kingdom (UK), the Medicines and Healthcare products Regulatory Agency (MHRA) granted marketing authorization for lecanemab^4^ and donanemab^5^ in 2024, while in Europe, approval was granted for both agents in 2025.^6^, ^7^ These regulatory decisions have heightened interest in whether early initiation of treatment alters longer‐term clinical outcomes beyond the 18‐month primary outcome of licensing trials and whether study data support evidence of disease modification, a fundamental goal in the field. This has become important, as measured short‐term benefits within the trial period are modest for the amyloid antibodies, and there are multiple AD therapeutic agents currently under clinical development,^8^ which will face future scrutiny.

Despite widespread use of the term, there is no universally established definition of what is meant by disease modification in the context of AD. One conceptual definition is of a therapy that results in a change in the underlying disease process that produces an enduring effect on the clinical course of AD.^9^ From a regulatory perspective, European Medicines Agency (EMA) guidance indicates that a therapy may be considered to have a disease‐modifying effect if it is shown to (1) delay underlying pathological or pathophysiological disease processes, and (2) this effect is accompanied by improvement in clinical signs and symptoms of the dementing condition.^10^

Regulators such as the U.S. Food and Drug Administration (FDA) approve therapies on the basis of evidence of benefit on pre‐specified clinical outcomes.^11^ As such, demonstration of statistically significant slowing of decline over a finite placebo‐controlled period may meet regulatory criteria for efficacy, but does not in itself establish disease modification. This differentiation is important clinically: a therapy may produce a short‐term symptomatic effect, without affecting subsequent progression, whereas disease modification would suggest an enduring change in trajectory.^12^ Yet, AD clinical trials have repeatedly struggled to distinguish between symptomatic and disease‐modifying effects.^12^, ^13^ Recent publications reporting long‐term data for lecanemab from open‐label extension (OLE) studies,^14^ and for donanemab using comparisons with external control cohorts,^15^ have highlighted the complexities clinicians face when evaluating whether the study design, analyses, and data are sufficient to support claims of disease modification. Planche et al.^16^ have specifically advocated for the use of delayed‐start trial designs when assessing anti‐amyloid therapies, particularly as a means of distinguishing symptomatic effects from true disease modification.

These developments further emphasize the need for a more structured framework for evaluating claims of disease modification in AD, which this Perspective seeks to provide. Specifically, this Perspective aims to equip clinicians with a framework for interpreting disease modification claims, outline the different trial designs that are used and analytical approaches used to test or support such claims, and proposes a practical appraisal checklist to evaluate studies.

### 1.1 What do Alzheimer's disease clinical trials really tell us about disease modification?

Most Phase III clinical trial designs used to evaluate AD therapies use a randomized, parallel‐group, placebo‐controlled trial over a pre‐specified period (e.g., 12–24 months)^17^ that is relatively short compared with overall illness duration.^16^ The primary trial objective is to demonstrate a statistically significant difference between drug and placebo groups at the trial endpoint on a pre‐specified cognitive and functional/global outcome.^17^

Biomarker evaluations, through the use of magnetic resonance imaging (MRI), amyloid–positron emission tomography (PET), and cerebrospinal fluid measurements are usually included to support evidence of target engagement and biological plausibility.^17^ However, the FDA published draft guidance in 2024 that established a reduction of brain amyloid burden as a surrogate efficacy endpoint.^18^ We would suggest that AD biomarker measures can only offer, at best, supportive evidence for disease modification. Concordance of clinical outcomes and biological measures can support biological plausibility. However, this concordance does not establish where the biomarker lies on the causal pathway of disease progression.^19^ In AD there is considerable uncertainty about the extent to which removal of amyloid in symptomatic individuals leads to a durable clinical benefit,^20^ or whether this occurs too late in the cascade to modify disease course.^21^ Mediation analyses have been proposed as a means of assessing the individual associations between biomarkers and clinical outcomes, including associations among amyloid PET load clearance, tau PET load dynamics, rate of medial temporal lobe atrophy, and Clinical Dementia Rating–Sum of Boxes (CDR‐SB) scores.^16^ Such analyses may help to determine whether downstream biomarker effects occur in parallel with sustained clinical benefit and strengthen the biological plausibility of disease modification.

Cognitive and functional composite endpoint‐based analyses are essential for establishing evidence of clinical efficacy. However, a statistically significant difference at one time point, even if maintained over follow‐up, does not distinguish between a symptomatic effect and an altered disease trajectory indicating disease modification.^9^ A therapy may exert an early improvement on a cognitive‐functional score, and then a subsequent decline can occur in a course that is parallel with placebo. Such continued separation would reflect a symptomatic benefit rather than disease modification^22^ and is what is typically reported for cholinesterase inhibitors in AD, such as in the Donepezil and Memantine in Moderate to Severe Alzheimer's Disease study (DOMINO) where participants who remained on donepezil treatment appeared to have a delayed transition to residential care.^23^

As a result, several analyses can be performed as potentially supportive evidence for disease modification, as they can also be affected by symptomatic therapies. These include slope analyses (to assess the change in rate of decline), increasing drug–placebo effect separation over time, and a delay in reaching a clinically meaningful milestone, for example, conversion to dementia or loss of functional independence.^17^

Of note, the interpretability of these analyses hinges on the trial design. Analyses derived from non‐randomized, unblinded, or non‐controlled studies will not be sufficiently robust to safely demonstrate such changes. For example, OLE studies, where participants complete an initial period of randomization to drug versus placebo and then are all switched to active treatment, will face issues of differential attrition, survival bias, and loss of blinding, all of which limit their ability to robustly support definitive claims of disease modification.^17^

### 1.2 Trial design to support claims of disease modification

Trial designs intended to support disease modification claims need to address the main limitation of a conventional, parallel‐arm trial: an inability to distinguish between symptomatic benefit and disease modification. Such a design should test whether early treatment results in an enduring advantage that cannot be recaptured by delayed initiation, that is, whether the delayed‐start group fails to “catch up” to those treated earlier.

FDA guidance states that a randomized‐start or withdrawal trial design represents the most convincing approach to demonstrating a persistent effect on disease course, with a randomized‐start design generally considered the most appropriate for use in AD.^24^, ^25^

In such a randomized or “delayed‐start” design, participants are randomized to either an early or delayed start group (Phase 1). At the end of a pre‐specified period, the early start group continues active treatment, whereas the delayed start group who were originally allocated to placebo is switched to active treatment, with blinding to Phase 1 treatment allocation maintained (Phase 2). Although there has been concern that such a trial design faces feasibility challenges, it has been implemented in other neurodegenerative disorders, for example, in The Attenuation of Disease Progression with Azilect Given Once‐daily (ADAGIO) trial.^22^ This prospectively assessed whether early initiation of treatment with rasagiline was advantageous over delayed start of treatment in Parkinson's disease, although the trial has faced criticism concerning methodological issues surrounding choice of pre‐specified non‐inferiority margin and randomization.^26^

The delayed start design enables direct comparison of treatment effects across phases by quantifying the between‐group difference at the end of Phase 2 (Δ_2_), relative to the difference observed at the end of Phase 1(Δ_1_). One approach is to then to pre‐specify a disease modification definition as a preservation margin of the Phase 1 effect (e.g., Δ_2_ ≥50% of Δ_1_), with control of type 1 error.^26^ The trial must be of sufficient duration to be able to detect meaningful change in disease trajectory. A trial duration of 18 months for the core randomized controlled trial (RCT) with an additional 18 months in the extension phase aligns with regulatory precedent for detecting meaningful changes in outcomes. The trial must also prospectively specify how potential sources of bias will be addressed, such as treatment discontinuation, treatment interruptions, drop‐out, and loss to follow‐up.

Accordingly, a delayed start design should prespecify at least two confirmatory questions. First, during Phase 1, superiority of active treatment over placebo must be demonstrated on a clinically meaningful outcome. Second, during Phase 2, the disease modification hypothesis must test whether delayed‐start participants fail to “catch up” with early‐start participants by more than a prespecified margin, with appropriate type 1 error control. This should be defined a priori with a defined margin (effect preservation).

Effect‐preservation analyses have limitations. The framework is endpoint‐based, relying on between‐group difference at set time points, rather than accounting for changes in disease progression trajectory or slope,^26^ and the choice of preservation threshold (e.g., 50%) is arbitrary.^9^ Furthermore, if participants with worse outcomes drop out more frequently (as usually happens in trials), the apparent preservation margin may reflect survival‐attrition bias^27^ rather than disease modification. As such, these analyses can help support claims of disease modification and should be interpreted in the context of the trial design, sensitivity analyses, and patterns of attrition.

There are challenges with a randomized delayed‐start trial design that should be acknowledged. First, if at the end of Phase 1, there is non‐superiority of treatment over placebo, this will not become apparent until after Phase 2 has been initiated, which will result in a trial continuing past the point of establishing non‐superiority. Second, as with any AD long‐duration trial, premature subject discontinuations and loss to follow‐up are likely to occur.^28^

Further challenges in interpretation arise when a drug may have both symptomatic and disease‐modifying effects. This issue was highlighted in the ADAGIO trial, where attempts were made to distinguish between rasagiline's established symptomatic effects and its putative disease‐modifying effects. A similar issue may apply to anti‐amyloid therapies, which also may exert dual action, an early symptomatic effect potentially through the removal of amyloid from synapses and a later disease‐modifying effect through alteration of the amyloid cascade.^16^ If we were to follow the principle of being able to identify both symptomatic and disease‐modifying effects explored in ADAGIO, this could entail a similar hierarchical multiple outcome approach, for example, (1) difference in slope between drug and placebo during initial drug‐placebo comparison, (2) difference in change in primary outcome from baseline to end of delayed start phase, and (3) difference in slope between early‐ and delayed‐starters during the delayed‐start phase. In a delayed‐start trial design, this combination would be expected to produce different patterns of results, depending on the presence and extent of disease‐modification or symptomatic benefit. A pure disease‐modification would show differences in the first two of the hierarchical outcomes but not the third. A purely symptomatic benefit would be accompanied by differences only in the first and third. Finally, a combination of disease modification and symptomatic benefit would lead to maintained differences on the first but attenuated differences on the second and third that might not reach accepted levels of statistical separation, depending on the relative contribution of symptomatic and disease‐modification to any differences. So, if the treatment effect is predominantly symptomatic, the second outcome is unlikely to remain significantly different between groups, whereas if mostly disease‐modifying, it will be the third outcome that fails to remain significantly different. Distinguishing these effects would require formal longitudinal analyses to assess the difference in slopes before and after delayed treatment initiation and overall change from baseline to the end of follow‐up.^26^

Nevertheless, when prospectively designed, adequately powered, and conducted under maintained blinding and randomization, a delayed‐start RCT remains the most robust approach for evaluating whether treatment effects persist beyond what would be expected from symptomatic benefit alone and for providing stronger evidence of a durable change in disease course. The key elements of such a trial design are presented in Summary Box 1.

**Summary Box 1: Key elements of delayed‐start trial design**

1. Prospectively designed, randomized, double‐blind, delayed‐start design
2. Pre‐specification of potential sources of bias (e.g., treatment discontinuation, treatment interruptions, drop‐out, loss to follow‐up)
3. Long enough trial duration to detect trajectory change
4. Clinically meaningful outcomes assessed at the end of both Phase 1 and Phase 2
5. Any predefined biomarker analysis plan is used as supportive evidence and not as a substitute for clinical outcomes

### 1.3 Alternative AD trial study designs

A withdrawal trial design, where participants are initially commenced on active treatment and subsequently randomized to either continue active treatment or switch to placebo, has also been proposed to test persistence of treatment effect. However, this approach has limitations, which make this design less desirable than a delayed‐start trial. These include uncertainty regarding how long treatment effects last after discontinuation, relapse of symptoms,^13^ and ethical concerns related to withdrawing a potentially clinically beneficial treatment.^28^

Longer‐duration, parallel‐group, double‐blinded, placebo‐controlled RCTs such as EXPEDITION and EXPEDITION2,^29^ which randomized treatment with intravenous solanezumab every 4 weeks, are examples of Phase III studies. These clinical trials took place over 80 weeks, maintained randomization and blinding over the duration, and included a prespecified statistical analysis plan. However, such trials are not designed to assess between a symptomatic and disease‐modifying effect and the authors accordingly make no claims regarding disease modification on the basis of endpoint separation.

EXPEDITION‐EXT was an open‐label extension (or OLE), offered to participants who completed EXPEDITION and EXPEDITION2 trials.^30^ All participants were assigned to open‐label treatment at the end of randomization, although patients and trial site personnel remained blinded to the original randomized assignment. Although “early‐start” versus “delayed‐start” comparisons were constructed from these data, these analyses are presented as exploratory, as randomization to treatment was not preserved beyond the placebo‐controlled phase and the extension phase was vulnerable to attrition and survivor bias. Only RCT completers who agreed to enroll would have been eligible to start the OLE, so they were a non‐randomized subset of the original randomized sample.

A much greater interpretative challenge arises when external control cohorts are used to make long‐term comparisons, such as in the TRAILBLAZER‐ALZ 2 long‐term extension of donanemab.^15^ Participants randomized to treatment continued on donanemab at the end of randomization in an additional 78‐week double blind extension (“early‐start” participants). Those participants who completed the placebo‐controlled period were started on donanemab in the extension (“delayed start” participants). Comparisons were made between early‐start and delayed‐start participants against an external Alzheimer's Disease Neuroimaging Initiative (ADNI) cohort. Such comparisons are limited by differences in study conduct and assessments, time periods and geographic regions, and residual or unmeasured confounding. These factors confound any causal interpretation of long‐term “early‐start” versus “delayed‐start” differences when using an external cohort.

The example of hydromethylthionine mesylate (HMTM; TauRx) further illustrates the interpretive risks associated with external control comparisons. Despite negative primary outcomes in a randomized, double‐blind, placebo‐controlled trial,^31^ subsequent analyses comparing treated participants with progression estimated from ADNI suggested apparent treatment benefit.^32^

Approaches including pre‐specification of matched external‐control cohorts and analytic plans, as well as replication across multiple external cohorts, alternative weighting strategies, and missing‐data sensitivity analyses, may strengthen confidence in OLE findings by testing whether results remain consistent under different assumptions. However, as these historical controls are not randomized, hidden confounding and residual bias remain possible despite statistical adjustment.

Table 1 presents a hierarchy of study designs, which have been ranked by their ability to differentiate between symptomatic and disease‐modifying treatment effects. The table is intended to help the clinician to align the strength of evidence from a study design against claims made of disease modification. The higher ranked designs preserve randomization and blinding over time and can more validly test the persistence of treatment effects. Lower ranked studies rely on descriptive analyses, post hoc analyses, or the use of external controls and are more subject to issues of confounding and bias. It is important to note that lower‐ranked studies can still provide valuable information regarding long‐term safety, tolerability, and hypothesis generation.

**TABLE 1:** Relative strength of clinical trial designs for evaluating disease modification.

Rank	Study design	Randomization	Blinding	What it can show	Limitations
1	Delayed‐start RCT	Y	Y	Durable treatment benefit formal test of failure to catch up	Sensitive to differential attrition and Missingness Not At Random (MNAR) missingness (missingness is related to patient outcomes)
2	Withdrawal RCT	Y	Y	Persistence of treatment effects after discontinuation	Persistence may reflect slow length of treatment effect (e.g., slow drug washout) or relapse of symptoms and ethical constraints
3	Long duration parallel‐group double‐blinded RCT	Y	Y	Sustained treatment benefit comparison of trajectories	Cannot distinguish early symptomatic effect from true trajectory change; no explicit test of catch up
4	Open‐label extension following RCT	N	N	Long term safety and tolerability	Loss of randomization and blinding, survivor bias, descriptive analyses
5	Historical or external control comparisons	N	N	Benchmarking	Confounding secondary to population differences and lack of controls
6	Post hoc subgroup or trajectory analyses	Variable	Variable	Exploratory analyses	Data selection, lack of pre‐specified hypotheses/error controls

### 1.4 How should data be analyzed and reported?

It is essential to initially clarify the estimand being addressed. An estimand provides a definition of what the trial treatment effect aims to estimate, and includes the population of interest, the time point of evaluation, and how intercurrent events are handled (e.g., discontinuation of treatment).^33^

Once the estimand is defined, appropriate statistical methods should be used to estimate it. Use of longitudinal modeling is important in AD trials as clinical outcomes are collected as repeated measures over time. An example of this is the Clinical Dementia Rating–Sum of Boxes (or CDR‐SB),^34^ which is assessed at multiple visits. Therefore, analyses should use a longitudinal model that estimates the between‐group differences (drug vs placebo), while also accounting for within‐patient correlation and incomplete follow‐up. One approach commonly used is the mixed model for repeated measures (MMRM).^35^ This estimates the adjusted mean trajectories across visits using available post‐baseline data and the missing‐at‐random (MAR) assumption.^36^

However, due to the longer duration of AD trials and the progressive nature of the disease, participant discontinuation and missing data are common and may occur in an informative way. For example, participants typically may discontinue treatment due to perception of clinical worsening. In this context, the MAR assumption may not be sufficient or correct. Sensitivity analyses should be pre‐specified and reported to account for any divergence from the MAR, such as scenarios consistent with missing‐not‐at‐random (MNAR) mechanisms.^37^

Consistent with the delayed‐start preservation framework described by Liu‐Seifert et al., disease‐modification inference should be based primarily on endpoint values rather than slope estimates alone.^30^ Therefore, the end of Phase 2 analyses in a randomized delayed‐start trial should include between‐group least‐squares (LS) mean differences (Δ_2_). This refers to the model‐estimated difference in the adjusted mean outcome between the early‐ and delayed‐start groups at a pre‐specified time point. This differs from an observed mean difference, as LS means are derived from the MMRM and account for baseline differences, repeated measures, and missing values. The Phase 2 LS Δ_2_ therefore represents the best estimate of the remaining treatment advantage after both groups have received active therapy and is used to assess whether there is a preserved early treatment benefit.

It may also be time to reconsider the most appropriate primary endpoint in delayed‐start AD trials. Continuous measures such as CDR‐SB can demonstrate statistically significant between‐group differences and meet requirements for licensing from the FDA or EMA, but the clinical meaning of small mean differences is often difficult for clinicians, patients, and caregivers to interpret. A more relevant endpoint, such as time to transition from a global CDR 0.5 to CDR 1, representing transition from mild cognitive impairment (MCI) to mild dementia, could be examined in time‐to‐event analyses. This transition would reflect a meaningful change in disease stage and may be more interpretable as clinically relevant. However, there are also limitations to this approach. Event rates are slow and may require longer trials with larger sample sizes, and the need to rely on potentially courser categorical staging transitions.^38^

It is essential that the clinician who is trying to evaluate a claim of disease modification is provided with numerical values from outcome measures as opposed to purely displays of graphical figures; suggested important elements of data reporting are presented in Summary Box 2.

**Summary Box 2: Key elements of delayed‐start trial data reporting**

1. The estimand is explicitly stated
2. Numeric tables of least‐square means and least‐square mean differences at each visit and corresponding 95% confidence intervals for between‐group differences
3. Transparent reporting of attrition by group, reasons for discontinuation, and results of sensitivity analyses

### 1.5 Examples of how to appraise an AD trial that claims disease modification

When evaluating a clinical trial that makes claims of disease modification, a clinician should consider the following questions:

### 1.6 Clinician's appraisal checklist

1. **Trial design**: Is the trial prospectively designed to test for disease modification (e.g., a randomized, double‐blinded, delayed‐start design?)
2. **Pre‐specified hypothesis**: Is there a pre‐specified disease‐modification testing hypothesis, for example, a defined effect preservation margin (non‐inferiority or partial preservation of Phase 1 effect)?
3. **Estimand**: Is the estimand clearly stated? This should include specification of the population, outcome, outcome time point, and handling of intercurrent events.
4. **Primary analyses**: Are differences between “early‐start” and “delayed‐start” groups reported numerically (e.g., LS mean differences), with corresponding 95% confidence intervals (CIs)? Are conclusions based on endpoint preservation rather than visual inspection of curves or slope estimates alone?
5. **Handling of missing data**: Are missing data and attrition adequately addressed with sensitivity analyses?
6. **Supportive analyses**: Are slope analyses or biomarker concentration changes framed as supportive evidence?
7. **Transparency of reporting**: Are sufficient numerical data provided to enable independent interpretation? Are limitations in study design and analyses acknowledged by the authors?

Published extension study data for lecanemab and donanemab are used as examples of applying the clinician's appraisal checklist to AD clinical trials and to illustrate examples where the study design, analyses, and reported data may be insufficient to robustly support claims of disease modification.

### 1.7 Lecanemab

Data from the Clarity AD 36‐month OLE were published in October 2025,^14^ and are appraised in Example 1.

Example 1 Clarity AD Open‐Label Extension (Lecanemab)

1. **Trial design**: This is an 18‐month OLE, following an 18‐month double‐blinded placebo‐controlled RCT (Core Study) of lecanemab versus placebo. At the end of the Core Study, all participants were subsequently switched to active drug for an 18‐month OLE (Extension phase). During the OLE, randomization and blinding have not been maintained. Those randomized to lecanemab in the core study are considered “early‐start” participants, and to placebo “delayed‐start” participants.
2. **Pre‐specified hypothesis**: There is no reported pre‐specified disease‐modification hypothesis. Analyses are reported as being descriptive.
3. **Estimand**: Participants were those that had completed the Core Clarity AD study. The endpoints included number of adverse events, changes in physical and laboratory tests, and change from core study baseline on composite measures such as the CDR‐SB. Additional endpoints included change in biomarker concentrations (change from core study baseline in amyloid PET using Centiloids). Efficacy was compared to an external ADNI cohort at 36 months. There is no information on handling of intercurrent events.
4. **Primary analyses**: Adjusted mean changes from baseline in CDR‐SB are presented graphically between the “early‐start” and “delayed‐start” groups and external ADNI cohorts over 36 months.
5. **Handling of missing data**: OLE study does not report on prespecified sensitivity analyses.
6. **Supportive analyses**: Time to worsening analysis (shift from MCI to dementia or mild dementia to moderate/severe dementia) presented.
7. **Transparency of reporting**: Primary analysis data are not provided in a numeric table (e.g., LS means and LS mean differences at each visit and corresponding 95% CIs for between‐group differences).

Analyses are framed as descriptive. Authors acknowledge that OLE phase is an open‐label, non‐randomized study. Limitations in using an external ADNI cohort, including difficulty in matching of baseline characteristics, are acknowledged. The loss of both blinding and randomized allocation after the Core phase means that any apparent group differences at 36 months may reflect confounding and expectancy effects rather than a true benefit of earlier treatment initiation.

### 1.8 Donanemab

Data from the long‐term extension (LTE) of the 76‐week TRAILBLAZER‐ALZ 2 trial of donanemab were published in December 2025^15^ and are appraised in Example 2.

Example 2 TRAILBLAZER‐ALZ 2 Long‐Term Extension (Donanemab)

1. **Trial design**: This is a 78‐week LTE, following on from a 76‐week double‐blinded placebo‐controlled RCT of donanemab versus placebo. At the end of the RCT, all participants were subsequently switched to active drug. During the LTE, participants and trial personnel remained blinded to prior randomization. Those randomized to donanemab in the RCT are considered “early‐start” participants, and to placebo “delayed‐start” participants in the LTE.
2. **Pre‐specified hypothesis**: There is no pre‐specified disease‐modification hypothesis for the LTE period. There is no effect‐preservation margin, non‐inferiority criterion, or formal hypothesis testing comparing early‐ and delayed‐start groups at the end of the LTE. Analyses are reported as exploratory.
3. **Estimand**: There is no stated estimand for the LTE analyses. The target population and handling of intercurrent events (e.g., treatment discontinuation) are not clearly defined.
4. **Primary analyses**: Efficacy in the LTE period, measured by change from baseline in CDR‐SB, is evaluated in the early‐ and delayed‐start groups against an external ADNI cohort. Numerical LS mean differences with corresponding 95% CIs are not presented for the difference between the early‐and delayed‐start groups at the end of the LTE. Data are only provided graphically as numerical trajectories.
5. **Handling of missing data**: Some descriptive handling of missing data is presented. No pre‐specified analyses for deviation from MAR assumptions, for example, MNAR.
6. **Supportive analyses**: Slope analyses and biomarker concentration changes (amyloid‐PET) are framed as supportive evidence.
7. **Transparency of reporting**: Primary analysis data are not provided in a numeric table. Authors acknowledge limitations, including, use of an external control cohort, with differences in study conduct, assessments, time periods, and geography. They also acknowledge baseline (at the end of the first 76 weeks) severity differences between the delayed‐start and early‐start participants, which may bias efficacy comparisons in favor of the early‐start group.

Despite the claim made in both papers that the treatment showed a disease‐modifying effect, this cannot be robustly supported by the LTE design or analyses.^39^, ^40^

## 2 CONCLUSIONS

There is great importance in adequately testing and supporting claims of disease modification because any drug that appears to disrupt the underlying pathology of AD carries the promise of changing the ultimate course of both neurodegeneration and clinical features, and modest short‐term benefits of treatment seen in 18‐month trials are surpassed by the promise of accruing long‐term advantages in terms of disability, reduced loss of independence, and maintained cognitive functioning. From a marketing perspective this is a highly potent, attractive message and as such there will be obvious post‐regulatory motivation from drug companies to show such effects. Claims of such long‐term benefits has immediate appeal to potential prescribers and the lay community, including prospective patients and their families.

Clinicians are gatekeepers of the communication of evidence and promise around the effects of treatment, and they carry a heavy responsibility to be accurate and fair in the information they disseminate. As we have demonstrated, the existing trial data do not safely substantiate claims of disease modification for lecanemab or donanemab. Unfortunately, the text of the papers reporting the OLE data for both drugs includes very definitive claims of disease modification, and clinicians will understandably go on to use this when they talk about treatment with patients and families.

In summary, AD studies to date do not provide strong evidence of disease modification. Neither the CLARITY AD OLE (lecanemab) data or the TRAILBLAZER‐ALZ 2 LTE (donanemab) are delayed‐start RCTs. Furthermore, neither study presents formalized statistical hypothesis testing or a pre‐specified effect preservation margin. Therefore, these studies are not structured to support causal claims of disease modification. Notwithstanding these limitations, the data that are presented do not support these claims. Primary efficacy outcomes are presented graphically and do not report tabular data with between early‐ and delayed‐start group LS mean differences and CIs. A visual inspection of the early‐ and delayed‐start groups in TRAILBLAZER‐ALZ 2 show that both groups worsened by around 4 points on the CDR‐SB independent of early or late initiation of donanemab, indicating that late‐start participants did catch up to early‐start ones.^41^ There are no reported sensitivity analyses to control for data biases, and both studies chose to compare early‐ and delayed‐start groups against an external ADNI cohort (with absence of most comparison demographic or missing data). Although data from both studies can be beneficial for informing safety and tolerability over extended periods, and exploratory hypothesis generation, findings should be reported responsibly and conservatively, noting the limitations of the study design, to ensure accurate interpretation by the scientific, clinical, and patient community.

We recommend that future studies use a randomized delayed‐start trial design to evaluate claims more robustly. We hope that this article will provide a scaffold to evaluate any future studies that make claims of disease modification and that this encourages clinicians to exercise necessary caution when interpreting such important causal claims.

## CONFLICT OF INTEREST STATEMENT

J.H. reports no disclosures relevant to the manuscript. K.Y.L. reports no disclosures relevant to the manuscript. R.H. reports unpaid advisory board work for Synaptogenix in the past 5 years. Author disclosures are available in the Supporting Information.

## CONSENT STATEMENT

There were no human participants in this study and therefore consent was not necessary.

## Supporting information




**Supporting Information**: alz71643‐sup‐0001‐ICMJE.pdf
